# Prevalence of Screening Patients for Alcohol Use in West Virginia: A Cross-Sectional Observational Study

**DOI:** 10.13023/jah.0703.04

**Published:** 2025-09-01

**Authors:** Bayan Abuhalimeh, Christopher Waters, R. Constance Wiener

**Affiliations:** West Virginia University; West Virginia University; West Virginia University

**Keywords:** alcohol use screening, alcohol use, alcohol, Appalachia, BRFSS, substance use, West Virginia

## Abstract

**Introduction:**

Consequences of alcohol consumption are well-established and compounded by physiological changes of aging (increased sensitivity; medication interactions; etc.). Alcohol screening for all ages is recommended, especially as most Americans believe moderate drinking is acceptable. Screening is important in high alcohol use regions, such as rural areas where isolation is more likely, especially for older adults. The Appalachian Region has a significant rural population of older adults. West Virginia (WV) is representative of Appalachia. It is a rural state, has many older adults, and all counties are Appalachian.

**Purpose:**

The purpose of this cross-sectional study is to determine the likelihood of alcohol screening among older versus younger WV adults.

**Methods:**

Behavioral Risk Factor Surveillance System 2022 WV data were used. Study inclusion was complete data on sex (at birth), age, alcohol use, and alcohol screening responses. Data were analyzed using Rao-Scott Chi square and logistic regression analysis for the association of age on alcohol screening at routine healthcare visits.

**Results:**

Of the entire sample (n=3,297), 30% were ≥65 years; 53% were female; and 35.7% reported moderate drinking. Alcohol screening was 58.4% for individuals ≥65 years, and 89.8% for individuals ages 18 to <40 years>( *p*<0.0001). Individuals ≥65 years were nearly 5 times more likely than individuals ages 18 to <40 years to not be screened (adjusted odds ratio=4.84 [95% CI: 3.24, 7.23]; *p*<0.0001).

**Implications:**

There is a need for greater screening for alcohol use in older adults, as older adults are disproportionately less likely to receive an alcohol screening compared to individuals <40 >years.

## INTRODUCTION

Alcohol consumption is common. It is an inherent aspect of many religious, cultural, and social traditions, as well as the most widely consumed recreational substance worldwide.^1^ However, it is the second-most misused substance in the United States (U.S.)^2^ The majority of adult Americans (84.1%) have had alcohol at least once in their lifetime.^3^

Alcohol over use can be problematic. It is associated with liver cirrhosis; various cancers; chronic pancreatitis; cardiovascular and respiratory systems disorders; renal diseases; urological pathologies; type 2 diabetes mellitus; infectious diseases; central nervous system disorders^4^; and cognitive decline attributing to dementia.^5^ In the U.S., approximately 6.2 million disability-adjusted life years are related to alcohol.^6^

Alcohol has significant health considerations specific to aging. Normal aging is associated with reduced muscle mass and lower body water content, which result in higher blood alcohol levels and slower alcohol metabolism in older adults compared to younger adults.^7^ Slower metabolism increases alcohol’s sleep-inducing sedative effect and impacts balance, coordination, and the likelihood of falls, which are often more severe than falls not associated with alcohol.^8^ Additionally, alcohol increases the negative side effects of many medications. Older adults are more likely to use medications that interact with alcohol such as analgesics, NSAIDs, sedatives, antidepressants, and antihistamines.^9^ Alcohol use in older adults is common. Current or heavy alcohol use was observed in 15.8% of older adults in one study.^10^

Older adults are at particular risk and should be screened for alcohol use. The U.S. Centers for Disease Control and Prevention (CDC) reported 38% of alcohol-related 2020 – 2021 deaths occurred in individuals aged ≥ 65 years.^11^ Despite recommendation for routine alcohol screening, screenings are often inadequately implemented and rarely conducted during primary care visits in the U.S.^12^

The older adult population is increasing. Older adults comprised approximately 17% of the U.S. population in 2020.^13^ One U.S. region with a significant older adult rural population is the Appalachian Region. The people in Appalachia face many health issues living in rural/remote or isolated areas. These include insufficient access and stigma toward alcohol-related concerns.^14^ The older adult population in Appalachia reached 17.6% in 2016, nearly four years ahead of the rest of the nation and continues to grow. WV is representative of Appalachia and the challenges to older adults living in rural Appalachia,^15^ including alcohol misuse.

Therefore, the purpose of this cross-sectional study is to assess the likelihood of alcohol screening among adults in WV at the last routine checkup (within the previous two years), comparing older adults (aged ≥65 years) to younger adults (aged 18 to <40 years). It is anticipated that there will be fewer screenings provided to the older adults than the younger adults. The null hypothesis is that there is no significant difference in the adjusted odds ratio of alcohol screening between older and younger adults.

## METHODS

This study has a cross-sectional observational study design. WV study data were extracted from the 2022 Behavioral Risk Factor Surveillance System (BRFSS) survey. The survey is conducted by the CDC as a health-related telephone survey. The collection design allows for the survey to be nationally representative of U.S. residents and their health-related risk behaviors, chronic health conditions, and use of preventive services.^16^ A complete description of the survey is available elsewhere.^16^

Participants included from the BRFSS 2022 for this study were WV adults >18 years. The specific question regarding alcohol screening that was asked by the interviewer in the BRFSS survey was: “You told me earlier that your last routine checkup was [within the past year/within the past 2 years]. At that checkup, were you asked in person or on a form if you drink alcohol?”^16^ The potential responses were yes or no. Other variables included complete data on sex at birth (male, female), age (18 to <40, 40 to <50, 50 to <60, 60 to <65, ≥65 with the age group 18 to <40 as the reference in the final model), alcohol use (a BRFSS calculated variable of: no use, moderate use [defined as ≤7 drinks/week for females or ≤14 drinks/week for males], heavy use [defined as >7 drinks/week for females or >14 drinks/week for males] in the past 30 days). Also included as potential confounders were race/ethnicity (non-Hispanic white, non-Hispanic black, other), smoking status (current, former, never), education (high school or less, some college/technical school/above), body mass index (<25, 25 to <30, ≥30), depression (yes, no), cancer (yes, no), cardiovascular disease (yes, no), diabetes (yes, no). Income and health insurance had significant missing values and were only presented as descriptors.

### Statistical Analysis

Data analyses were conducted with SAS® version 9.4 (SAS, Cary, NC) (SAS, 2025). A significance level of <0.05 was established *a priori*. For the comparison of screened versus not screened bivariate comparisons, Rao-Scott Chi-square tests (for complex survey design) were used to assess the association of age with alcohol screening. The BRFSS provided the weights, clusters, and strata to allow for generalization from the sample. Logistic regression analysis was conducted to examine the association of age on screening for alcohol use at the routine checkup visit, assessed by unadjusted odds ratio (UOR), adjusted odds ratio (AOR) and 95% Confidence Intervals (CI) controlling for the significant associations resulting from the bivariate analysis.

### Ethics Statement

The research received acknowledgement as non-human subject research by the West Virginia University Institutional Review Board (2111472366).

## RESULTS

Table 1 presents the characteristics of the individuals in the study. Among the 3,297 individuals from WV, 30% were ≥65 years; 53% were female; 90% were white; and 22.8% had an education > high school. There were 5.2% who reported heavy drinking; and 35.7% who reported moderate drinking. Alcohol screening was reported by 76.3%.

In Rao-Scott Chi-square analysis (Table 2), when age was dichotomized at <65 and ≥65 years, 83.5% of individuals ages <65 years reported being screened for alcohol use at their last routine checkup and 59.4% of individuals ≥65 years were screened. Similar relationships exist among the stratified age groups with less likelihood of screening as age increased (*p*<0.001). Other significant factors for being less likely to be screened were a high school education or less (*p*<0.001), having a lower income (*p*=0.0003), having cancer (*p*<0.001), having cardiovascular disease (*p*<0.001), or having diabetes (*p*<0.001). Participants with depression (*p*=0.0157), and heavy alcohol use (*p*<0.001) were more likely to be screened.

Table 3 presents the unadjusted and adjusted logistic regression of age on not having been screened. Compared to the age group of participants ages 18 to <40 years, participants ≥65 years were six times more likely to not be screened *p*<0.0001 (Unadjusted odds ratio = 6.01; 95% CI: 4.16, 8.68). Of note, though not the focus of the study, age groups 50 to <60 years, and 60 to <65 years were also more likely to not be screened (*p*<0.0001 for the groups). In adjusted logistic regression, adjusted for the significant factors from the Chi-square analysis (education, income, depression, cancer, cardiovascular disease, diabetes, and alcohol use) there were similar results. Participants ages ≥65 years had an AOR of 4.84 (95% CI: 3.24, 7.23; *p*<0.0001) of not being screened as compared with those ages 18 to <40 years; that is, they were approximately five times more likely to not receive alcohol screening as compared to the comparison group of individuals ages 18–40 years. Participants ages 60 to <65 had an AOR of 3.34 (95% CI: 2.13, 5.23; *p* <0.0001) of not being screened as compared with those ages 18 to <40 years. The age group with participants 50 to <60 years had an AOR of 1.99 (95% CI: 1.30, 3.04; *p*=0.0015) as compared with the age group of participants 18 to <40 years of not having been screened. It should be noted that an analysis for the interaction of sex and age failed to reach significance, and sex was not included in the final model.

Data that were not presented in a table format are the relationship of age and alcohol use among the WV sample. The reports of heavy drinking were 6.9% for ages 18 to <39; 6.3% for ages 40 to <50; 6.5% for ages 50 to <60; 5.2% for ages 60 to <65; and 2.2% for ages ≥65 (*p*<0.0001). The reports of moderate drinking were 47.6%, 40.6%, 35.2%, 33.9%, and 23.1%, respectively (*p*<0.0001).

## IMPLICATIONS

Alcohol screening is an important part of identifying vulnerable individuals and making early interventions. In this study, a majority of individuals ≥65 years were not screened for alcohol use at their regular medical check-ups (58.4%). WV individuals ≥65 years were less likely than younger adults to be screened on alcohol-related issues. Adults ≥65 years had an AOR of 4.84 (95% CI: 3.24, 7.23; *p*<0.0001) of not receiving screening compared to the adults ages 18 to <40 years. This reveals a lack of reported screening and therefore a greater need for it among WV’s older population as older adults are at heightened risk for alcohol-related health issues when alcohol is misused.

Overall, of the 3,297 WV adults (≥18 years) in this study, 5.2% reported heavy drinking, and 35.7% reported moderate drinking. The 5.2% statistic for heavy drinking is similar to the results from the U.S. National Health Interview Survey (NHIS) in which 5.1% of adults reported heavy drinking; however, the moderate drinking result of the current study (32.7%) is much higher than the 15.5% national level.^17^

The result for heavy drinking among WV adults ages ≥65 years (2.2%) was lower than the NHIS; however, the moderate drinking level (23.1%) for WV older adults was much higher than the 15.5% national level. These findings are consistent with surveys of older adults, which show an alarming prevalence of older adults at risk of harmful drinking in spite of the well-documented negative health consequences of alcohol use in this age group.^18^ As the aging population increases, the public health implications of alcohol misuse in this age group become more salient.

Other researchers also indicated older individuals are less likely to be screened for alcohol use compared to younger individuals.^19^ Mauro and colleagues^20^ reported that among older adults who consumed alcohol and used the healthcare system in the past year, 24.68% of men and 27.04% of women lacked any alcohol screening or counseling. Since drinking alcoholic beverages exposes older people to higher health risks it is essential that health professionals screen and advise these groups of individuals on alcohol consumption on a consistent basis^21^ to avoid adverse consequences for health and/or increased healthcare costs.^22^

Though not the focus of the study, WV individuals who reported depression or heavy drinking were more likely to be screened, which suggests that healthcare providers may prioritize screening for individuals with more apparent or severe alcohol-related symptoms.^23^ Other variables influencing the likelihood of alcohol screening in WV included lower education, lower income, and chronic illness such as cardiovascular disease and diabetes. These conditions tend to overlap with healthcare access and quality, ultimately affecting the likelihood of alcohol related evaluations.^24^

### Study strengths and limitations

One of the key strengths of this study is its use of the 2022 BRFSS data from WV, which provides a large, population-based sample that is representative of WV adults. This enhances the generalizability of the findings to similar rural U.S. states with a significant population of older adults. Additionally, the study employs robust statistical analyses, including Rao-Scott Chi-square tests and logistic regression models, to assess the relationship between age and alcohol screening. These methods account for potential confounders and strengthen the validity of the findings. Furthermore, by focusing on an at-risk demographic (older adults in a state with a high proportion of individuals aged ≥65 years) this study highlights an important public health issue that has been historically overlooked.

However, the study also has limitations that should be considered when interpreting the results. First, the use of self-reported data introduces the potential of recall and social desirability biases, as participants may either underreport or overreport their alcohol consumption or screening experiences. Second, while the BRFSS is a well-established surveillance tool, it does not capture detailed clinical data or confirmatory medical records that could provide additional context on alcohol screening practices. The binary nature of the screening question (“yes” or “no”) also limits insights into the quality, frequency, and effectiveness of these screenings. Additionally, potential residual confounding may exist despite statistical adjustments, as unmeasured variables such as provider attitudes, healthcare access, and patient willingness to disclose alcohol use could influence screening rates.

### Policy Recommendations

There is a need for targeted measures, including improved alcohol screening for older adults in Appalachia, where alcohol-related health risks are amplified by demographic and regional factors. Routine alcohol use screening for older adults is crucial to identify at-risk individuals early and to implement brief interventions or referrals to treatment as necessary. The U.S. Preventive Services Task Force’s recommendation statement is for primary care providers to screen for unhealthy alcohol use in adults ≥18.^25^ Healthcare providers may benefit from enhanced training on the importance of alcohol screening in older adults. Incorporating alcohol screening prompts into electronic health records may help ensure that screenings are conducted routinely during medical visits. Additionally, public health campaigns aimed at older adults and their caregivers should be strengthened, focusing on education, raising awareness, and encouraging the disclosure of misuse to better manage alcohol-related issues that may arise.

The results of this study highlight a number of major issues regarding alcohol consumption and screening among older adults. The evidence suggests alcohol misuse may be a covert condition in older WV individuals. With increased awareness and reduced stigma regarding alcohol intake, it is possible to enhance more honest interaction between healthcare workers and patients, which would lead to the identification of persons at risk at an earlier stage.^19^

Ultimately, while the authors shed light on the disparity in alcohol screening rates between younger and older adults, they do not explore the underlying reasons. Future researchers should consider qualitative approaches or provider-focused studies to better understand these barriers.

Longitudinal studies would be beneficial in tracking changes over time and assessing the impact of interventions aimed at improving alcohol screening rates among older adults. Despite these limitations, this study provides critical evidence supporting the need for increased alcohol screening effort with aging populations.

**SUMMARY BOX**

**What is already known about this topic?**

WV is representative of Appalachia and the challenges to older adults living in rural Appalachia,^15^ including alcohol misuse.

**What is added by this report?**

Alcohol misuse may be a covert condition in older WV individuals. There is a need for greater screening for alcohol, particularly for older adults.

**What are the implications for future research?**

There is a need for targeted measures, including improved alcohol screening for older adults in Appalachia, where alcohol-related health risks are amplified by demographic and regional factors. Routine alcohol use screening for older adults is crucial to identify individuals who are at risk early and to implement brief interventions or referrals to treatment as necessary.

## Figures and Tables

**Table 1:** Study Characteristics of Participants, BRFSS, 2020, n= 3297

	Number	Weighted %
**Sex**		
Female	1899	53.0
Male	1398	47.0
**Age**		
18 – <40	540	28.0
40 – <50	427	14.8
50 – <60	576	17.2
60 – <65	364	10.0
≥65	1390	30.0
**Race/Ethnicity**		
Non-Hispanic white	3107	90.0
Non-Hispanic black	68	3.5
Other	122	6.4
**Smoking Status**		
Current	571	19.6
Former	965	27.9
Never	1761	52.5
**Education**		
HS or less	2191	77.2
Some college	N/A	N/A
Or above	1106	22.8
**Health Insurance**		
Yes	3233	96.9
No	64	3.1
**Body Mass Index**		
<25	786	23.4
25 – <30	1108	32.7
≥30	1403	43.8
**Income**		
<$25,000	581	18.1
≥ $25,000	1728	52.4
Missing	988	29.0
**Depression**		
Yes	944	30.4
No	2353	69.5
**Cancer**		
Yes	734	17.8
No	2563	87.2
**Cardiovascular Disease**		
Yes	499	12.8
No	2798	87.3
**Diabetes**		
Yes	720	19.6
No	2577	80.4
**Alcohol Use**		
Heavy	144	5.2
Moderate	1128	32.7
No use	2025	59.1
**Alcohol screening**		
Yes	2414	76.3
No	883	23.7

NOTES: HS = high school

**Table 2:** Associations of Alcohol Screening with Variables of Interest, BRFSS 2022, n= 3297

	Screened		Not Screened			
	Number	Weighted %	Number	Weighted %	*p*-value	degrees of freedom
**Sex**					0.1753	1
Female	1385	75.1	514	24.9		
Male	1029	77.5	369	22.5		
**Age (Years)**					<0.0001	4
18 to <40	487	89.8	53	10.2		
40 to <50	366	85.4	61	14.6		
50 to <60	460	79.7	116	20.3		
60 to <65	260	69.6	104	30.4		
≥65	841	59.4	549	40.6		
**Age (Years), Dichotomized**					<0.0001	1
<65	1573	83.5	334	16.5		
≥65	841	59.4	549	40.6		
**Race/Ethnicity**					0.7477	2
Non-Hispanic white	2276	76.4	831	23.6		
Non-Hispanic black	49	71.6	19	28.4		
Other	89	76.3	33	23.7		
**Smoking Status**					0.4523	2
Current	432	78.1	139	21.0		
Former	695	74.8	270	25.2		
Never	1287	76.3	883	23.7		
**Education**					<0.0001	2
HS or less	1535	74.2	656	25.8		
Some college			227	16.7		
Or above	879	83.3	874	24.0		
**Health Insurance**					0.2504	1
Yes	2359	76.0	874	24.0		
No	suppressed	suppressed	suppressed	suppressed		
**Body Mass Index**					0.2284	2
<25	554	73.5	232	26.5		
≥ 25 to < 30	816	77.4	292	22.7		
≥ 30	1044	77.0	359	23.0		
**Income**					0.0003	2
< $25,000	395	69.8	186	30.2		
≥ $25,000	1264	77.3	464	22.7		
Missing	755	78.4	233	26.8		
**Depression**					0.0157	1
Yes	719	79.5	225	20.5		
No	1695	74.8	658	25.2		
**Cancer**					<0.0001	1
Yes	493	67.3	241	32.7		
No	1921	78.2	642	21.8		
**Cardiovascular Disease**					<0.0001	1
Yes	333	65.1	166	34.9		
No	2081	77.9	717	22.1		
**Diabetes**					<0.0001	1
Yes	472	65.9	248	34.1		
No	1942	78.8	635	21.2		
**Alcohol Use**					<0.0001	2
Heavy	123	87.7	21	12.3		
Moderate	941	30.6	187	14.4		
No use	1350	69.6	675	30.4		

NOTES: HS = high school

**Table 3:** Logistic Regression of Age on Not Having an Alcohol Screening, BRFSS 2022, n= 3297

	Unadjusted Odds Ratio (95% CI; *p-*value)	Adjusted Odds Ratio (95% CI; *p-*value)
**Age**		
18 to <40	Reference	Reference
40 to <50	1.41 (0.94, 2.40; 0.0865)	1.99 (1.30, 3.04; 0.0015)
50 to <60	2.24 (1.48, 3.41; <.0001)	1.99 (1.30, 3.04; 0.0015)
≥65	6.01 (4.16, 8.68; <.0001)	4.84 (3.24, 7.23; <.0001)
**Education**		
HS or less		1.33 (1.06, 1.67; 0.0146)
Some college or above		Reference
**Income**		
< $25,000		1.29 (0.98, 1.70; 0.714)
≥$25,000		Reference
Missing		1.14 (0.89, 1.45; 0.3097)
**Depression**		
Yes		0.86 (0.69, 1.11; 0.2745)
No		Reference
**Cancer**		
Yes		0.95 (0.75, 1.19; 0.6411)
No		Reference
**Cardiovascular Disease**		
Yes		0.98 (0.73, 1.33; 0.9144)
No		Reference
**Diabetes**		
Yes		1.19 (0.93, 1.51; 0.1720)
No		Reference
**Alcohol Use**		
Heavy		0.47 (0.26, 0.86; 0.0145)
Moderate		9.52 (0.41, 0.66; <.0001)
No use		Reference

NOTES:

1 The model was controlled for the significant relationships determined in the Chi-square analysis.

The unadjusted and adjusted models had 3293 degrees of freedom.

Abbreviations: HS = high school
